# Oral health-related quality of life in Northland Māori children and adolescents with Polynesian amelogenesis imperfecta

**DOI:** 10.3389/fdmed.2024.1485419

**Published:** 2024-11-22

**Authors:** Michelle Martin, Sunitha Gowda, Lyndie Foster Page, W. Murray Thomson

**Affiliations:** ^1^ Oral Health Service Te Tai Tokerau, Hospital and Specialist Services, Health New Zealand | Te Whatu Ora, Whangārei, Northland, New Zealand; ^2^ Division of Dental Public Health, School of Dentistry, Oregon Health & Science University, Portland, OR, United States; ^3^ Department of Oral Sciences, Faculty of Dentistry, The University of Otago, Dunedin, New Zealand

**Keywords:** Polynesian AI, Poly AI, amelogenesis imperfecta, Māori, oral health-related quality of life

## Abstract

**Introduction:**

Amelogenesis Imperfecta (AI) is a hereditary developmental disorder of tooth enamel with few known variants with differing characteristics, depending on where in the amelogenesis process an error has occurred. Polynesian AI (or Poly AI) is prevalent among people of Polynesian descent including New Zealand Māori. While the impact of AI on the quality of life has been reported in some studies, the role of Poly AI on oral health-related quality of life (OHRQoL) is not known. This study explores OHRQoL among New Zealand Māori with and without AI.

**Methods:**

A cross-sectional study was undertaken, with ethical approval obtained from the New Zealand Health and Disability Ethics Committee. 30 Māori children and adolescents with Poly AI, and 60 age and sex matched Māori children and adolescents with no Poly AI (as the comparison group) were randomly selected and recruited to participate in the study. OHRQoL was measured using the 19-item COHIP-SF.

**Results:**

Statistically significant differences were observed in the OHRQoL between those with Poly AI and the comparison group. Linear regression analyses controlling for age and deprivation showed significantly poorer OHRQoL among those with Poly AI than in those with no Poly AI.

**Discussion:**

The study findings highlight poorer OHRQoL among Māori children with Poly AI, emphasizing the need for early detection and management of the condition and the importance of providing appropriate training in diagnosing Poly AI and managing hypersensitivity. Further research among Polynesian populations is needed to understand the impact of Poly AI on OHRQoL.

## Introduction

Amelogenesis imperfecta (AI) is a hereditary developmental disorder of tooth enamel which has a number of known variants with differing characteristics, depending on where in the amelogenesis process an error has occurred (1–5). The clinical appearance of AI can differ remarkably among types (6). For example, *hypoplastic* AI is characterised by a quantitative defect of the enamel, whereby it may be thinner, pitted or grooved but is adequately mineralised. *Hypo-mature* AI is defined by poorly mineralised brittle enamel due to incomplete removal of protein from the enamel matrix. *Hypocalcified* AI enamel is inadequately mineralised due to insufficient transport of calcium ions into the developing enamel.

**Figure 1:**
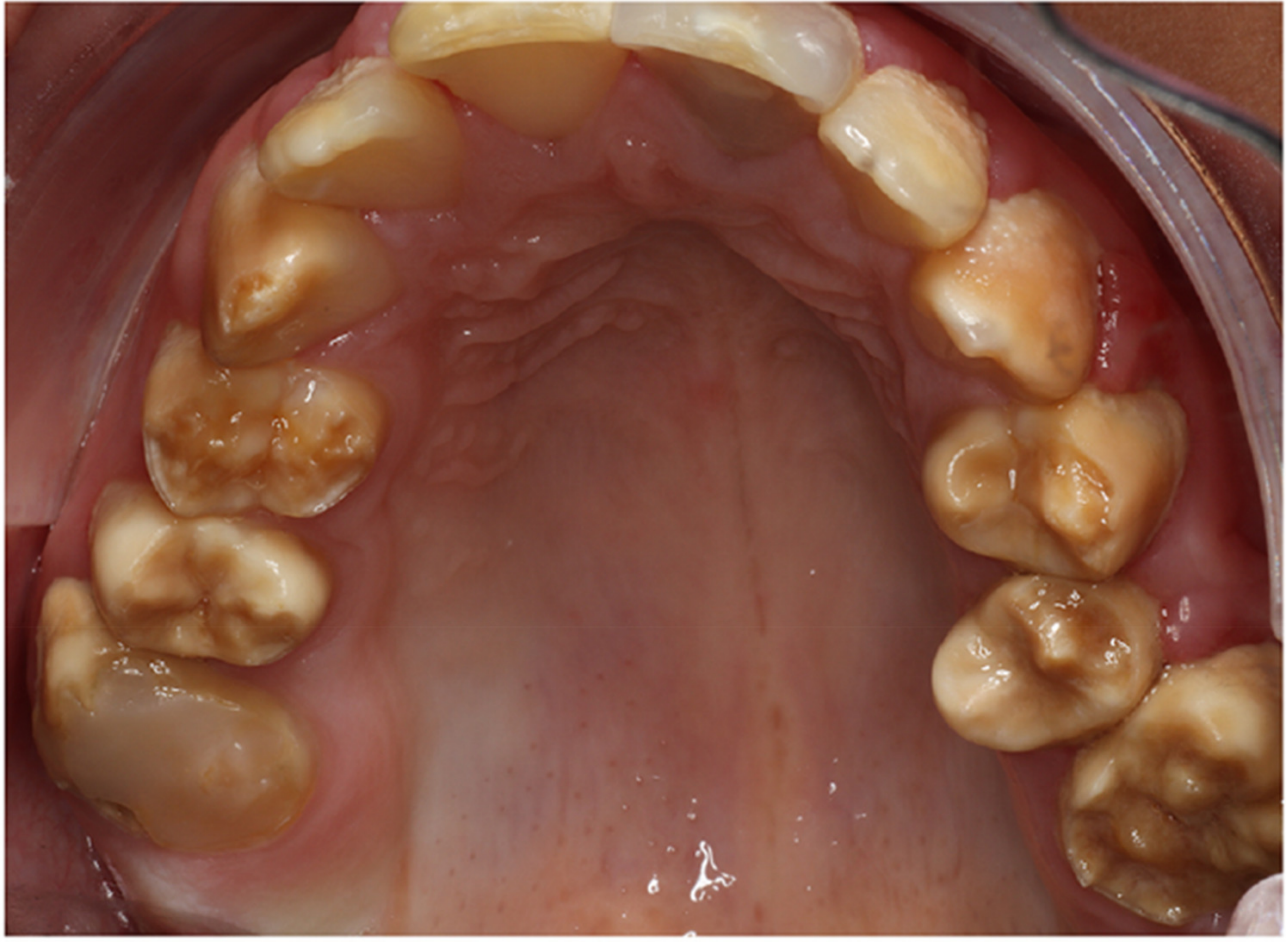
A typical Poly AI patient with maxillary hypomineralised dentition exhibiting mottled yellow and brown discolouration.

**Figure 2:**
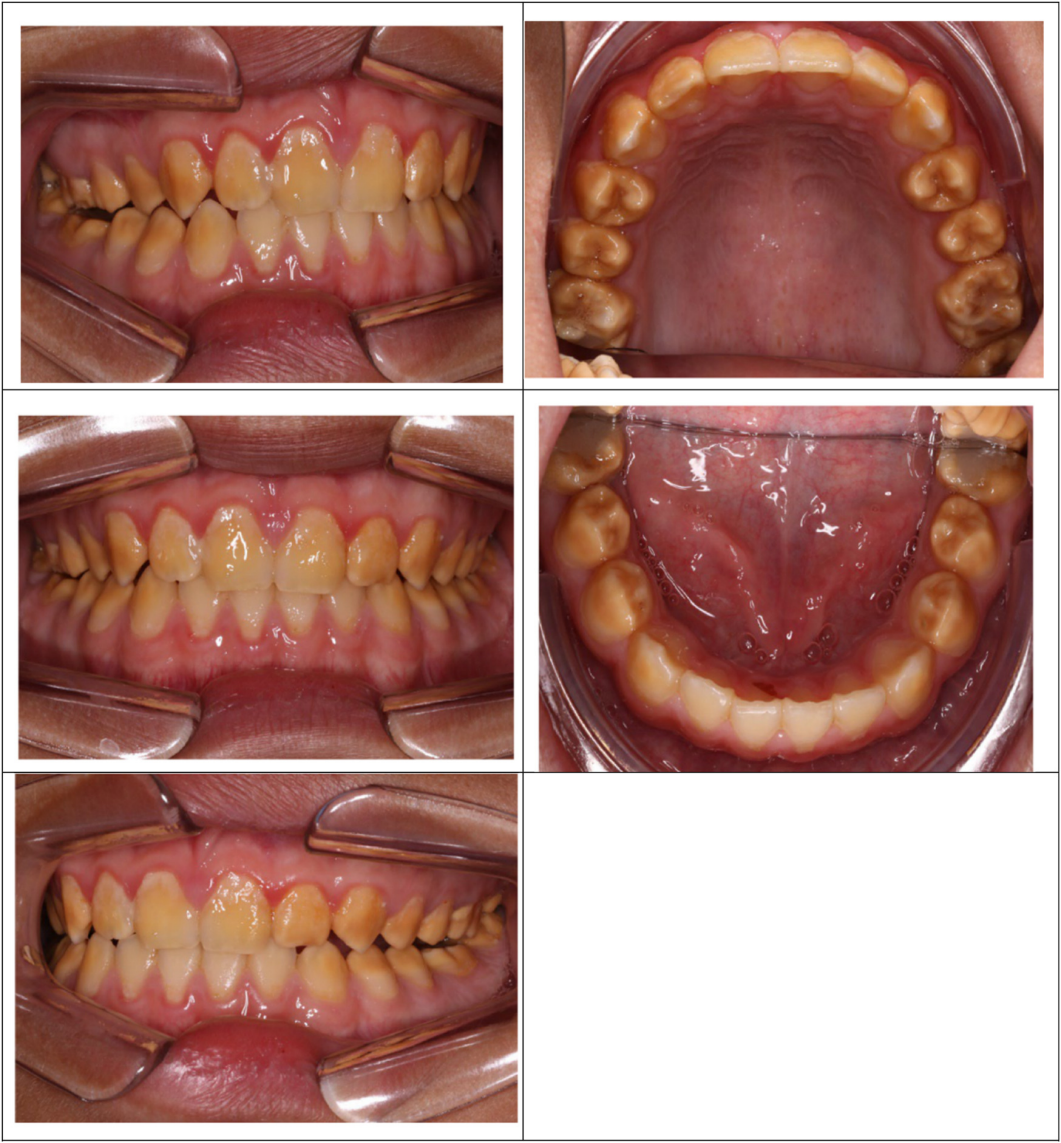
Poly AI case showing bilaterally symmetrical hypo-mineralised dentition, with mottled uniformly yellow to brown discoloration and an anterior to posterior gradient in severity.

A cluster of Māori children in Northland (New Zealand) are known to have a moderate-to-severe hypo-maturation form of AI known as “Polynesian Amelogenesis Imperfecta” (or Poly AI) which is found in people of Polynesian ancestry, including New Zealand Māori (6–9). Poly AI is characterised by a bilaterally symmetrical hypo-mineralised dentition, mottled or uniformly yellow to brown in colour, with an anterior to posterior gradient in severity. Figures 1, 2 show typical Poly AI cases. Its prevalence in the wider New Zealand Māori population and other Polynesian populations is unknown. Its occurrence in the primary dentition is also poorly understood, possibly due to limited phenotypic expression in that dentition. The chalky nature of the teeth predisposes to post-eruptive breakdown and hypersensitivity with a high associated treatment burden and poorer oral health. While some of the anterior dentition may be only mildly affected, this is not always the case, and the canines and premolars (which are within the smile line) can have poor aesthetics.

There has been limited research to date on the impact of AI, but that which has been reported has shown impacts on quality of life in people with AI in a number of domains, with pain, impaired ability to maintain oral hygiene, and effects on social interactions, confidence, self-esteem, and anxiety (2, 10–15). Sufferers experience a larger treatment burden and there are implications for the wider family (16–23).

Oral health has been defined by Locker as “a standard of the oral tissues which contributes to overall physical, psychological and social well-being by enabling individuals to eat, communicate and socialise without discomfort, embarrassment or distress and which enables them to fully participate in their chosen social roles” (24). Oral health-related quality of life (OHRQoL) measures have been designed with a biopsychosocial health approach to capture information on people's symptoms, physical functioning, and emotional and social well-being. AI in particular has been shown to affect OHRQoL. Children and adolescents with AI have concerns about aesthetics and function, as well as a high level of concern about comments by other people (13, 15). Adult patients with AI have substantially poorer OHRQoL than people without the condition (11); a recent systematic review described sufferers' concerns about aesthetics, hypersensitivity, function, and adverse effects on well-being and social interaction (17). Following treatment of AI-affected teeth using crowns in 69 Swedish children and adolescents, a considerable improvement in OHRQoL was observed, with the mean OHIP-14 score falling from 8.8 (sd, 5.9)–2.0 (sd, 2.5), giving a large effect size of 1.2 (14).

Poly AI has received little attention within New Zealand or internationally. This is perhaps due to its rarity, the poor understanding of the needs of the children and adults who have it, and the overall lack of knowledge of this form of AI. Moreover, its impact on OHRQoL has not been examined. Accordingly, the aim of this study was to investigate OHRQoL in New Zealand Māori children with Poly AI and to compare it with that of their peers.

## Materials and methods

This was a cross-sectional study. Māori Health professionals and the area Māori Health Directorate were engaged in study co-design to ensure its credibility and acceptability to Māori. Ethical approval was granted by the New Zealand Health and Disability Ethics Committee (Ref: 18/CEN/143).

Children (tamariki) and adolescents (rangatahi) were screened by Northland Community Oral Health Service Dentists and Oral Health Therapists between April 2019 and November 2020, in order to identify cases of Poly AI. All dentists and oral health therapists were trained in how to identify cases but no formal calibration or intra-examiner measurements were recorded. Inclusion was based on the clinical features of the dentition defined as characteristic of Poly AI by the lead investigator. The criteria for identifying Poly AI participants included patients with bilaterally symmetrical hypomineralised dentition, exhibiting mottled or uniformly yellow to brown discoloration, with a severity gradient increasing from anterior to posterior teeth. Some 45 Māori tamariki and rangatahi were identified as having Poly AI. All of the identified individuals with Poly AI under 18 years of age were invited to participate. This was followed by a mail-out of study participant information and consent forms. Of the 45 with Poly AI, five were uncontactable and ten did not attend the clinical appointment for the study, leaving 30 cases (66.7%). All of the AI participants had received minimal dental treatment by the time of the survey.

Comparison children (matched by date of birth and sex) were selected from Northland Community Oral Health Service appointment books throughout the region. For each child with Poly AI seen at a specific dental clinic, a list of children with the same or closest date of birth and sex was generated from the appointment books. At the time of their dental visit, the child and parent/caregiver (if present) were approached by the study leads in waiting rooms, and discussion took place about the study face to face. Most of the children and parents/caregivers approached this way agreed to take part in the study. Written consent and child assent was obtained for all participants. Each participant was given a unique identification number used instead of his/her name to maintain confidentiality.

Sociodemographic information on the participants (age and sex) was collected. Neighbourhood deprivation was determined using an area-based deprivation measure (25) which allocated each participant to a deprivation decile score, based on the child's residential address. Areas with scores 1–3 were classified as “low deprivation”; those with scores 7–10 were classified as “high deprivation”.

OHRQoL was measured using the 19-item COHIP-SF, completed by all participants with assistance from trained research assistants if needed. The COHIP-SF comprises the three domains of *oral health*, *functional well-being* and *socio-emotional well-being* (26). For each of the 19 items, participants were asked how frequently they had experienced it relating to their teeth, mouth or face. Response options and scores were: “Never” (scoring 0); “Almost never” (1); “Sometimes” (2); “Fairly often” (3), and “Almost all of the time” (4). The COHIP-SF contains items to assess both positive and negative aspects of OHRQoL. At the analysis stage in this study, the negative items were reversed so that a higher score reflected greater OHRQoL. Two global oral health questions were used, in order to allow checking of the COHIP-SF's concurrent validity. First, children were asked to rate the health of their teeth, lips, jaws and mouth (response options: “Very good”, “Good”, “OK” or “Poor”). Second, they were asked how much their teeth, lips, jaw or mouth affect their life overall (response options: “Not at all”, “A little bit”, “Some” or “A lot”).

### Statistical analyses

Analyses were conducted using SPSS (version 28.0.1.0 for Windows). Following the computation of descriptive statistics for all variables, the internal consistency reliability of the COHIP-19 scale and its subscales was assessed using Cronbach's alpha, after which the COHIP-SF scale and subscale scores were computed. Bivariable comparisons for demographic characteristics used cross-tabulations and Chi-square tests in Table 1 to investigate (and demonstrate to the readers) whether there were systematic differences between the AI group and the Normal enamel group in those three important sociodemographic characteristics. The concurrent validity of the COHIP-SF scale and subscales was examined against responses to the two global oral health items, with the mean scale scores examined across the five ordinal response categories of each of the global items. Following confirmation of validity, the mean scale scores were examined by demographic characteristics and AI status. Finally, linear regression was used to model the (normally distributed) COHIP-SF scale and subscale scores, to determine the extent of the difference in scores between those with AI and those without, controlling for age and deprivation.

**Table 1:** Sociodemographic characteristics by dental enamel status (brackets contain column percentages unless otherwise indicated).

	Dental enamel status		Both combined
	Amelogenesis Imperfecta	Normal	
Sex			
Male	12 (40.0)	24 (40.0)	36 (40.0)
Female	18 (60.0)	36 (60.0)	54 (60.0)
Age group			
7–10	2 (6.7)	10 (16.7)	12 (13.3)
11–13	9 (30.0)	28 (46.7)	37 (41.1)
14–19	19 (63.3)	22 (36.7)	41 (45.6)
NZDep category			
Highly deprived	27 (90.0)	40 (66.7)^a^	67 (25.6)
Other	3 (10.0)	20 (33.3)	23 (74.4)
All combined^b^	30 (33.3)	60 (66.7)	90 (100.0)

^a^ *P* < 0.05.

^b^ Row% in brackets.

## Results

One-third of the sample comprised members with Poly AI, while the remainder were in the comparison group (Table 1). Females outnumbered males, and almost two-thirds of the AI group were aged 14–19 years, whereas just over one-third of the comparison group were. While two-thirds of the comparison group resided in highly deprived areas, almost all of the AI group did.

Mean COHIP-19 scores showed ascending gradients across the ordinal response categories of the two global items (Table 2), indicating the scale's acceptable concurrent validity in this sample. The pattern with the mean COHIP-19 subscale scores was largely similar, although it was less consistent for the Functional Well-being subscale scores by the impact of oral health on quality of life.

**Table 2:** Concurrent validity: mean COHIP-19 scores by self-reported oral health and impact on quality of life (brackets contain standard deviations).

	Mean COHIP-19	Mean well-being subscale scores		
		Oral health	Functional	Socio-emotional
Self-reported oral health				
Excellent	59.8 (13.1)^a^	16.2 (3.1)^a^	13.5 (2.7)^a^	30.1 (10.6)^a^
Very good	59.8 (8.0)	13.9 (2.8)	12.8 (2.4)	33.1 (4.2)
Good	50.7 (12.0)	11.7 (4.0)	11.6 (2.9)	27.5 (7.5)
Fair	43.4 (11.8)	10.0 (4.1)	11.6 (1.8)	22.2 (8.1)
Poor	31.5 (22.0)	8.5 (3.1)	7.0 (3.9)	16.0 (15.5)
Impact of oral health on quality of life				
Not at all	59.0 (11.4)^a^	13.6 (3.8)	12.6 (2.8)^a^	32.8 (5.9)^a^
Very little	55.0 (7.5)	12.6 (3.3)	11.8 (2.2)	30.7 (5.0)
Some	50.5 (12.5)	12.3 (4.5)	12.0 (2.8)	26.2 (6.6)
A lot	49.6 (15.4)	11.5 (4.2)	12.4 (3.6)	25.7 (10.3)
Very much	36.1 (18.3)	9.6 (4.5)	9.0 (3.0)	17.5 (13.3)
All combined	52.1 (13.5)	12.3 (4.0)	11.9 (2.9)	28.0 (8.6)

^a^ *P* < 0.05; Kruskal-Wallis *H* test.

Mean COHIP-19 scores showed consistent gradients by age group, whereby they were highest among the youngest and lowest among the oldest (Table 3); again, the gradient was less consistent with the Functional Well-being subscale scores. There were no differences by sex or deprivation category. There were marked, consistent differences in scale and subscale scores between the AI group and those with normal enamel, whereby the mean COHIP-19 scores were considerably lower among the former.

**Table 3:** Mean COHIP-19 scores by AI status and sociodemographic characteristics (brackets contain standard deviations).

	Mean COHIP-19	Mean well-being subscale scores		
		Oral health	Functional	Socio-emotional
Sex				
Male	53.4 (12.9)	12.8 (4.3)	12.2 (2.6)	28.4 (7.4)
Female	51.3 (13.9)	11.9 (3.9)	11.6 (3.1)	27.7 (9.4)
Age group				
7–10	56.3 (9.4)^a^	14.1 (4.1)^a^	12.0 (2.4)	30.2 (5.6)
11–13	55.2 (12.6)	13.2 (4.0)	12.3 (2.4)	29.8 (7.7)
14–19	48.1 (14.4)	10.9 (3.7)	11.5 (3.4)	25.8 (9.6)
NZDep category				
Highly deprived	52.9 (13.1)	12.1 (3.7)	11.7 (3.4)	29.0 (7.3)
Other	51.9 (13.6)	12.3 (4.2)	11.9 (2.7)	27.6 (9.0)
Enamel status				
AI	42.2 (13.8)^a^	9.6 (4.2)^a^	10.5 (3.6)^a^	22.1 (8.9)^a^
Normal	57.1 (10.2)	13.6 (3.3)	12.6 (2.3)	30.9 (6.8)

^a^ *P* < 0.05; Mann-Whitney *U* test where there are 2 categories; Kruskal-Wallis *H* test where there are 3 categories.

The linear regression analyses (Table 4) showed that, after controlling for age and deprivation, the COHIP-19 score was considerably lower among those with AI. Similar patterns were observed for the subscale scores.

**Table 4:** Summary of linear regression models for the COHIP-19 overall and subscale scores.

	B (95% CI)	*P* value
COHIP-19 overall score		
AI case	−14.34 (−19.69, −8.98)	<0.001
Age	−0.88 (−1.76, −0.002)	0.05
Highly deprived	2.65 (−3,00, 8.31)	0.35
Constant	66.72 (53.97, 79.46)	<0.001
Adjusted *R*^2^ = 0.292		
COHIP-19 *Oral health well-being* score		
AI case	−3.75 (−5.39, −2.12)	<0.001
Age	−0.37 (−0.63, −0.10)	0.008
Highly deprived	1.14 (−0.59, 2.86)	0.35
Constant	17.56 (13.66, 21.45)	<0.001
Adjusted *R*^2^ = 0.271		
COHIP-19 *Functional well-being* score		
AI case	−2.18 (−3.48, −0.87)	0.001
Age	−0.05 (−0.26, −0.17)	0.65
Highly deprived	0.81 (−0.58, 2.19)	0.25
Constant	12.65 (9.54, 15.76)	<0.001
Adjusted *R*^2^ = 0.098		
COHIP-19 *Socio-emotional well-being* score		
AI case	−8.41 (−11.96, −4.86)	<0.001
Age	−0.47 (−1.05, 0.12)	0.12
Highly deprived	0.71 (−3.05, 0.12)	0.71
Constant	36.51 (28.05, 44.97)	<0.001
Adjusted *R*^2^ = 0.236		

## Discussion

We compared OHRQoL in a group of New Zealand Māori children with and without Poly AI, finding that those with the condition had substantially poorer OHRQoL than their unaffected peers. The difference was particularly marked in the *Oral health* and *Socio-emotional* domains of the COHIP-19, and older children were more severely affected whereas no gender differences were observed, consistent with the findings in other studies (10, 11).

One of the strengths of the study is its focus on a relatively rare and unknown condition, Poly AI among New Zealand Māori children and providing valuable insights into the poorer OHRQoL of those with Poly AI than that of their peers without AI. Furthermore, the use of the COHIP-19 subscales enabled detailed assessment across the three domains of *oral health wellbeing*, *functional wellbeing* and *socio-emotional wellbeing.*

However, the study has several limitations. First, the participant numbers were limited by the condition's relative rarity in the population, but our sample size was similar to those of other studies which have assessed AI's quality of life impacts in children and adults (10, 11, 13, 14). Despite the relatively small sample size, there were statistically significant differences, suggesting that the investigation was indeed adequately powered; a *post hoc* power analysis based on comparing means with the observed effect size of 1.1 (the difference in mean COHIP score, 14.9, divided by the overall standard deviation, 13.5) gives a required N of 19 in each group for 95% power to detect a difference. Second, while the comparison group children were of a slightly younger age range by date of birth, they were of similar age at the time of data collection, given that the data collection for the comparison group had been delayed by the Covid pandemic. Lastly, that many of the participants lived in highly deprived areas might have influenced the findings, given the poorer OHRQoL which is usually observed in such neighbourhoods. This highlights the need for further research with people with AI from a broader range of deprivation levels.

In the *oral health* domain, when asked specifically about pain, two-thirds of children (tamariki) and adolescents (rangatahi) with AI reported often experiencing pain, while just over one-third of their unaffected peers did so. Given the high levels of dental disease among the unaffected peers, the AI group's chronic pain levels are a concern. Proper management of hypersensitivity before and during treatment is crucial to prevent exacerbating anxiety and the avoidance of dental care which includes the risk of pulpal trauma from chronic inflammation.

That the *socio-emotional* domain showed greatest impact was similar to the findings by Hashem et al. (11). Many patients reported experiences such as teasing, bullying and a lack of empathy from others. These issues are particularly concerning for children entering their teenage years, a developmental epoch when social pressures are heightened. Treatment intervention at an early age may reduce the negative impacts on their wellbeing, as demonstrated by Pousette Lundgren et al. (14, 16) and Chen et al. (22).

Impacts via *functional impairment* were less marked than the other two domains, possibly because the comparison group also had high dental caries experience and chronic dental pain.

The academic literature in New Zealand highlights oral health disparities between Māori and non-Māori children (27–29), with Māori experiencing poorer oral health. Māori children without AI have twice the number of decayed, missing, and filled teeth of non-Māori children, placing those with AI at even greater risk of poorer oral health. Additionally, 90% of the AI children in our study came from the most deprived households, suggesting considerable challenges to obtaining care to improve their OHRQoL. Individuals with Poly AI present with severe enamel defects, increased dental decay, functional impairments, and aesthetic concerns requiring comprehensive dental treatment often difficult to access.

Parekh et al. and Pousette Lundgren et al. found improved OHRQoL after treatment in children with AI, using both quantitative and qualitative research methods (13, 14). Their studies demonstrated that early and appropriate treatment interventions can significantly improve the quality of life for children with AI. This should include improved visibility of Poly AI within the education of oral and healthcare professionals to provide early screening, treatment and referral options for patients who present with Poly AI. In particular, these findings support the need for policies focused on the oral health of indigenous populations who present with Poly AI and policies to support training of culturally appropriate care for patients with Poly AI to mitigate the impacts on their overall quality of life.

The New Zealand Māori view of health recognises its holistic nature via four important and interconnected aspects: *taha tinana* (physical wellbeing), *taha hinengaro* (mental and emotional wellbeing), *taha wairua* (spiritual wellbeing), and taka whanau (social and family wellbeing) (27). Quality of life impacts for children and adolescents with AI will likely extend beyond their physical needs, impacting all aspects of their wellbeing. New Zealand Māori are a subset of a broader group of people of Polynesian descent with different cultural identities and life experience. Further research is needed to determine whether other groups with Polynesian ancestry who have this genetic condition (such as Tahitians, Samoans, Tongans, Cook Island Māori) have similar impacts on their quality of life and to determine the extent of improvement in OHRQoL following masking of the AI enamel with veneers or crowns. Qualitative research could also deepen understanding of how living with Poly AI affects these children and improve knowledge of how best we might help them.

## Conclusion

Our findings highlight the considerable impact of Poly AI on the OHRQoL of New Zealand Māori children, and they further stress the need for starting treatment interventions at an early age, raising awareness of the diagnosis and management of the condition among dental professionals, and managing hypersensitivity before and after treatment procedures. Greater public and professional awareness of the condition and its impact is needed, along with adequate and appropriate funding for preventive and aesthetic dental care for this group, in order to reduce the condition's impact.

## Data Availability

The raw data supporting the conclusion will be made available by the authors upon request, without undue reservation, though permission from the Hauora Māori Service Directorate, Health New Zealand | Te Whatu Ora, will be required before sharing or releasing any data related to Indigenous people.

## References

[1] Crawford PJ Aldred M Bloch-Zupan A. Amelogenesis imperfecta. Orphanet J Rare Dis. (2007) 2:17. 10.1186/1750-1172-2-17 17408482 PMC1853073

[2] Poulsen S Gjorup H Haubek D Haukali G Hintze H Lovschall H. Amelogenesis imperfecta—a systematic literature review of associated dental and oro-facial abnormalities and impacts on patients. Acta Odontol Scand. (2008) 66:193–9. 10.1080/00016350802192071 18615322

[3] Gadhia K McDonald S Arkutu N Malik K. Amelogenesis imperfecta: an introduction. Br Dent J. (2012) 212:377–9. 10.1038/sj.bdj.2012.314 22538897

[4] Smith C Poulter J Antanaviciute A Kirkham J Brookes S Inglehearn C Amelogenesis imperfecta; genes, proteins, and pathways. Front Physiol. (2017) 8:435. 10.3389/fphys.2017.00435 28694781 PMC5483479

[5] Bloch-Zupan A Rey T Jimenez-Armijo A Kawczynski M Kharouf N, O-Rare consortium: de La Dure-Molla M, et al. Amelogenesis imperfecta: next-generation sequencing sheds light on Wiktop’s classification. Front Physiol. (2023) 14:1130175. 10.3389/fphys.2023.1130175 37228816 PMC10205041

[6] Smillie AC Rodda JC Kawasaki K. Some aspects of hereditary defects of dental enamel, including some observations on pigmented Polynesian enamel. N Z Dent J. (1986) 82:122–5. 3461345

[7] Rodda JC Palamara J Phakey PP Smillie AC. The polarized light microscopy and ultrastructure of Polynesian pigmented tooth enamel. Arch Oral Biol. (1989) 34:475–81. 10.1016/0003-9969(89)90127-1 2597040

[8] Crooks MC. Prevalence of developmental defects of enamel in children and young adults in the Cook Islands. N Z Dent J. (1990) 86:39–41. 2371001

[9] Smillie AC Rodda JC Young D. The protein of pigmented Polynesian dental enamel. Arch Oral Biol. (1993) 38:717–24. 10.1016/0003-9969(93)90012-b 8215996

[10] Coffield KD Phillips C Brady M Roberts MW Strauss RP Wright T. The psychosocial impact of developmental dental defects in people with hereditary amelogenesis imperfecta. JADA. (2005) 136:620–30. 10.14219/jada.archive.2005.0233 15966649

[11] Hashem A Kelly A O’Connell B O’Sullivan M. Impact of moderate and severe hypodontia and amelogenesis imperfecta on quality of life and self-esteem of adult patients. J Dent. (2013) 41:689–94. 10.1016/j.jdent.2013.06.004 23778130

[12] Sneller J Buchanan H Parekh S. The impact of amelogenesis imperfecta and support needs of adolescents with AI and their parents: an exploratory study. Int J Pediatr Dent. (2014) 24:409–16. 10.1111/ipd.12086 24404886

[13] Parekh S Almehateb M Cunningham S. How do children with amelogenesis imperfecta feel about their teeth? Int J Pediatr Dent. (2014) 24:326–35. 10.1111/ipd.12080 24283507

[14] Pousette Lundgren G Wickstrom A Hasselblad T Dahloff G. Amelogenesis imperfecta and early restorative crown therapy: an interview study with adolescents and young adults on their experiences. Plos One. (2016) 11:e0156879. 10.1371/journal.pone.0156879 27359125 PMC4928800

[15] Lyne A Parekh S Patel N Lafferty F Brown C Rodd H Patient reported outcome measure for children and young people with amelogenesis imperfecta. Br Dent J. (2021) 6:3329–9. 10.1038/s41415-021-3329-9 PMC8420961 34489543

[16] Pousette Lundgren G Hassleblad T Johansson A Dahllof G. Experiences of being a parent to a child with Amelogenesis imperfecta. Dent J. (2019) 7:17. 10.3390/dj7010017 PMC6473584 30744129

[17] Appelstrand SB Robertson A Sabel N. Patient-reported outcome measures in individuals with amelogenesis imperfecta: a systematic review. Eur Arch Paediatr Dent. (2022) 23(6):885–95. 10.1007/s40368-022-00737-3 35896941 PMC9750902

[18] Lindunger A Smedberg J. A retrospective study of the prosthodontic management of patients with amelogenesis imperfecta. Int J Prosthodont. (2005) 18:189–94. 15945303

[19] Koruyucu M Bayram M Tuna EB Gencay K Seyman F. Clinical findings and long-term managements of patients with amelogenesis imperfecta. Eur J Dent. (2014) 8:546–52. 10.4103/1305-7456.143640 25512739 PMC4253114

[20] Ortiz L Pereira AM Jahangiri L Choi MJ. Management of amelogenesis imperfecta in adolescent patients: clinical report. J Prosthodont. (2019) 28:607–12. 10.1111/jopr.13069 31054208

[21] Lafferty F Al Siyabi H Sinadinos A Kenny K Mighell AJ Monteiro J The burden of dental care in amelogenesis imperfecta paediatric patients in the UK NHS: a retrospective, multi-centred analysis. Eur Arch Paediatr Dent. (2021) 22:929–36. 10.1007/s40368-021-00638-x 34146252

[22] Chen C Hu J Estrella M Peters M Bresciani E. Assessment of restorative treatment of patients with amelogenesis imperfecta. Pediatr Dent. (2013) 4:337–42. PMC5724038 23930633

[23] Quandalle C Baillot A Fournier B Garrec P de La Dure-Molla M Kerner S. Gingival inflammation, enamel defects, and tooth sensitivity in children with amelogenesis imperfecta: a case-control study. J Appl Oral Sci. (2020) 28:e20200170. 10.1590/1678-7757-2020-0170 32997085 PMC7521421

[24] Locker D. Measuring oral health: a conceptual framework. Community Dent Health. (1988) 5:3–18. 3285972

[25] Atkinson J Salmond C Crampton P. NZDep2018 Index of Deprivation, Interim Research Report. Wellington: University of Otago (2019).

[26] Broder HL Wilson-Genderson M Sischo L. Reliability and validity testing for the child oral health impact profile-reduced (COHIP-SF 19). J Public Health Dent. (2012) 72(4):302–12. 10.1111/j.1752-7325.2012.00338.x 22536873 PMC3425735

[27] Broughton JR. Te niho waiora me te iwi Māori:dental health and the Māori people. NZ Dent J. (1993) 89(395):15–8. 8441509

[28] Ministry of Health. New Zealand Health Strategy (2016). http://www.moh.govt.nz/nzhs.html (accessed March 23, 2024).

[29] Lacey JK Thomson WM Crampton P Willing E. Working towards Māori oral health equity: why Te Tiriti o Waitangi needs to underpin the oral health system, using evidence from the New Zealand oral health survey. NZ Dent J. (2021) 117(3):105–10.

