# Correction: Oral health-related quality of life in Northland Māori children and adolescents with Polynesian amelogenesis imperfecta

**DOI:** 10.3389/fdmed.2025.1711860

**Published:** 2025-10-31

**Authors:** Michelle Martin, Sunitha Gowda, Lyndie Foster Page, W. Murray Thomson

**Affiliations:** ^1^ Oral Health Service Te Tai Tokerau, Hospital and Specialist Services, Health New Zealand | Te Whatu Ora, Whangārei, Northland, New Zealand; ^2^ Division of Dental Public Health, School of Dentistry, Oregon Health & Science University, Portland, OR, United States; ^3^ Department of Oral Sciences, Faculty of Dentistry, The University of Otago, Dunedin, New Zealand

**Keywords:** Polynesian AI, Poly AI, amelogenesis imperfecta, Māori, oral health-related quality of life

A Correction on Oral health-related quality of life in Northland Māori children and adolescents with Polynesian amelogenesis imperfecta By Martin M, Gowda S, Foster Page L, Thomson WM. (2024). Front. Dent. Med 5:1485419. doi: 10.3389/fdmed.2024.1485419

**Error in figure/table**

Wrong content

There was a mistake in Figure 1 as published. The image of the maxillary arch has been positioned upside down. The corrected Figure 1 appears below.

**Figure 1:**
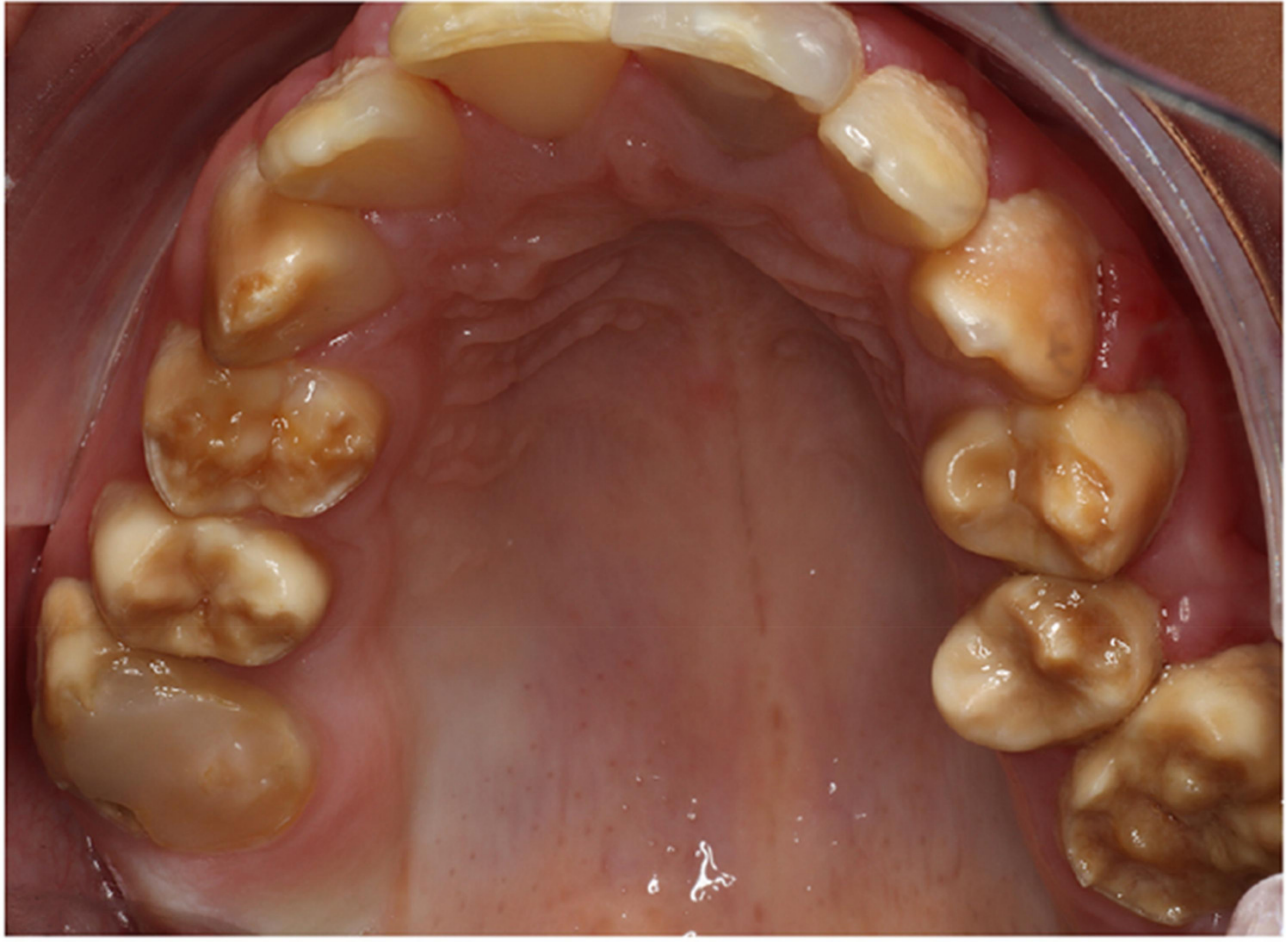
A typical Poly AI patient with maxillary hypomineralised dentition exhibiting mottled yellow and brown discolouration.

There was a mistake in Figure 2 as published. The images of the maxillary arch and mandibular arch have been positioned upside down and in the wrong order. The corrected Figure 2 appears below.

**Figure 2:**
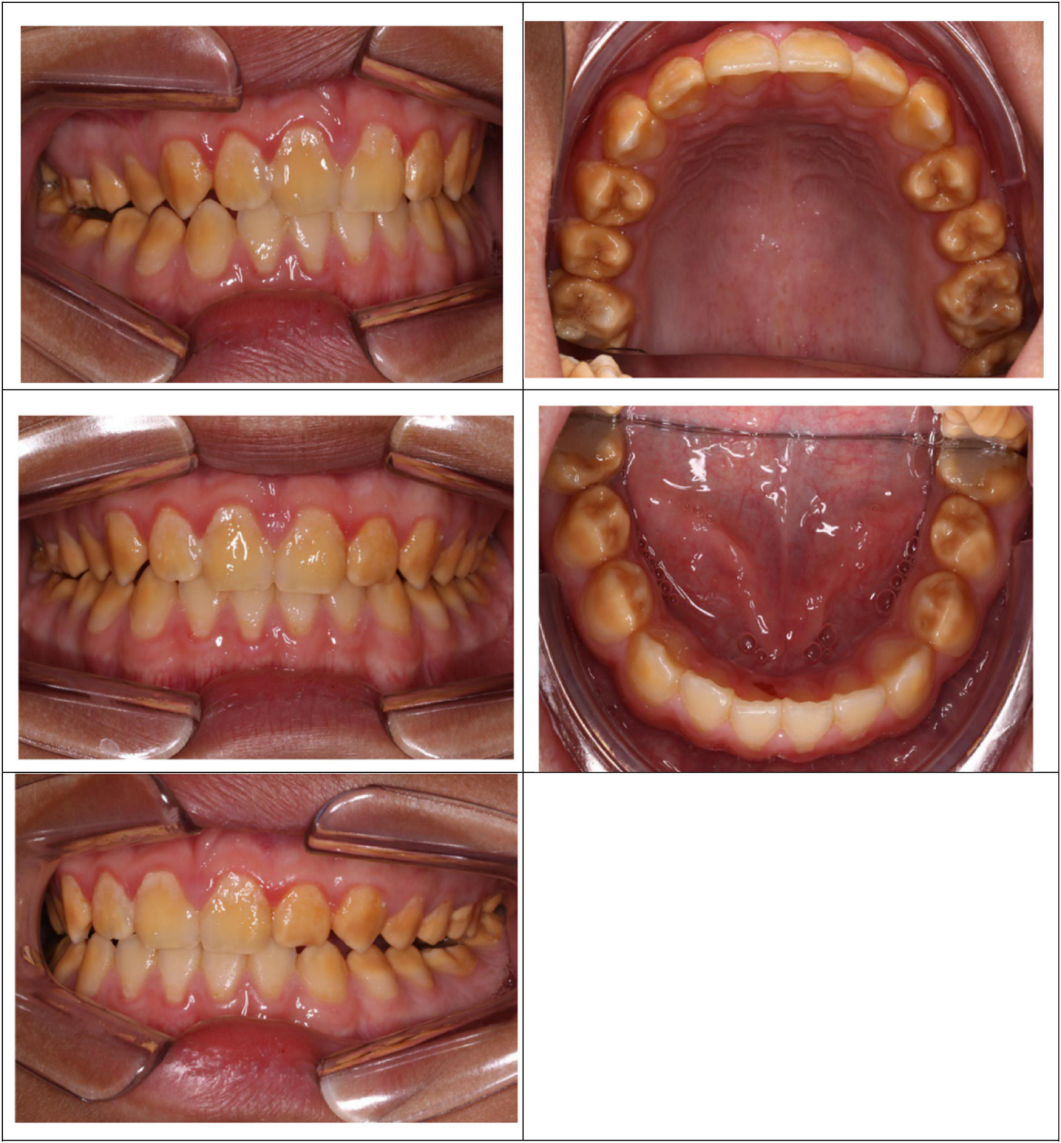
Poly AI case showing bilaterally symmetrical hypo-mineralised dentition, with mottled uniformly yellow to brown discolouration and an anterior posterior gradient in severity.

**Abstract**

Adding/removing text

In the abstract (the Introduction), an incorrect sentence was provided. This has been corrected to read:

Polynesian AI (or Poly AI) is prevalent among people of Polynesian descent including New Zealand Māori. While the impact of AI on the quality of life has been reported in some studies, the role of Poly AI on oral health-related quality of life (OHRQoL) is not known.

The original version of this article has been updated.

Adding/removing text

In the abstract (the Methods), an incorrect sentence was provided. This has been corrected to read:

30 Māori children and adolescents with Poly AI, and 60 age and sex matched Māori children and adolescents with no Poly AI (as the comparison group) were randomly selected and recruited to participate in the study.

The original version of this article has been updated.

Adding/removing text

In the abstract (the Discussion), an incorrect sentence was provided. This has been corrected to read:

Further research among Polynesian populations is needed to understand the impact of Poly AI on OHRQoL.

The original version of this article has been updated.

